# The State of Ambient Air Quality in Two Ugandan Cities: A Pilot Cross-Sectional Spatial Assessment

**DOI:** 10.3390/ijerph120708075

**Published:** 2015-07-15

**Authors:** Bruce J. Kirenga, Qingyu Meng, Frederik van Gemert, Hellen Aanyu-Tukamuhebwa, Niels Chavannes, Achilles Katamba, Gerald Obai, Thys van der Molen, Stephan Schwander, Vahid Mohsenin

**Affiliations:** 1 Division of Pulmonary Medicine, Department of Medicine, Makerere University College of Health Sciences, Kampala, Uganda; E-Mail: brucekirenga@yahoo.co.uk; 2 Department of Environmental and Occupational Health and Center for Global Public Health, School of Public Health, Rutgers University, NJ 07107, USA; E-Mails: mengqi@sph.rutgers.edu (Q.M.); schwansk@sph.rutgers.edu (S.S.); 3 Department of General Practice, University Medical Center Groningen, University of Groningen, Groningen 9700 RB, The Netherlands; E-Mails: frgemert@xs4all.nl (F.G.); t.van.der.molen@umcg.nl (T.M.); 4 Department of Paediatrics and Child Health, Makerere University College of Health Sciences, Kampala, Uganda; E-Mail: hellen.aanyut@gmail.com; 5 Department of Public Health and Primary Care, Leiden University Medical Center, Leiden 2333 ZA, The Netherlands; E-Mail: n.h.chavannes@lumc.nl; 6 Clinical Epidemiology and Biostatics Unit, Department of Medicine, Makerere University College of Health Sciences, Kampala, Uganda; E-Mail: akatamba@yahoo.com; 7 Department of Physiology, Faculty of Medicine, Gulu University, Gulu, Uganda; E-Mail: lekobai@gmail.com; 8 Department of Medicine, Yale University School of Medicine, New Haven, CT 06510, USA; E-Mail: vahid.mohsenin@yale.edu

**Keywords:** ambient air pollution, particulate matter, nitrogen dioxide, sulfur dioxide, ozone, Uganda, Kampala, Jinja

## Abstract

Air pollution is one of the leading global public health risks but its magnitude in many developing countries’ cities is not known. We aimed to measure the concentration of particulate matter with aerodynamic diameter <2.5 µm (PM_2.5_), nitrogen dioxide (NO_2_), sulfur dioxide (SO_2_), and ozone (O_3_) pollutants in two Ugandan cities (Kampala and Jinja). PM_2.5_, O_3_, temperature and humidity were measured with real-time monitors, while NO_2_ and SO_2_ were measured with diffusion tubes. We found that the mean concentrations of the air pollutants PM_2.5_, NO_2_, SO_2_ and O_3_ were 132.1 μg/m^3^, 24.9 µg/m^3^, 3.7 µg/m^3^ and 11.4 μg/m^3^, respectively. The mean PM_2.5_ concentration is 5.3 times the World Health Organization (WHO) cut-off limits while the NO_2_, SO_2_ and O_3_ concentrations are below WHO cut-off limits. PM_2.5_ levels were higher in Kampala than in Jinja (138.6 μg/m^3^ *vs.* 99.3 μg/m^3^) and at industrial than residential sites (152.6 μg/m^3^ *vs.* 120.5 μg/m^3^) but residential sites with unpaved roads also had high PM_2.5_ concentrations (152.6 μg/m^3^). In conclusion, air pollutant concentrations in Kampala and Jinja in Uganda are dangerously high. Long-term studies are needed to characterize air pollution levels during all seasons, to assess related public health impacts, and explore mitigation approaches.

## 1. Introduction

On the 25 March 2014, the World Health Organization (WHO) released new estimates of the contribution of air pollution to global mortality showing that seven million deaths were attributable to air pollution worldwide in the year 2012 (3.7 million due to ambient air pollution (AAP) and 4.3 million due to indoor air pollution (IAP)) [1]. This number represents a doubling from the air pollution mortality rates estimated by WHO in the year 2004 [1,2].

Air pollution is thus one of the leading global public health risks. Health problems commonly associated with air pollution exposure include: respiratory diseases (e.g., chronic obstructive pulmonary disease, asthma, lung cancer and acute respiratory infections in children) and cardiovascular diseases (such as ischemic heart disease and stroke) [2]. Adverse health effects associated with air pollution exposure are particularly severe among vulnerable populations (e.g., people with respiratory diseases like asthma), older people, and children. Available data also show that airpollutionhasthepotential to impair lung growth as a result of perinatal exposures thus threatening the health of entire generations [3,4,5,6]. Although over 3000 substances are known to potentially contaminate air [7], the WHO has identified particulate matter (PM), nitrogen dioxide (NO_2_), carbon monoxide (CO), sulfur dioxide (SO_2_) and ozone (O_3_) as the pollutants with greatest public health importance [2]. The United States (US) National Ambient Air Quality Standard (NAAQS) [8] designates all of the above plus airborne lead (Pb) as criteria pollutants.

WHO and the US Environmental Protection Agency (USEPA) have defined guideline limits for these pollutants that should not be exceeded in order to maintain and protect public health [9,10]. The WHO limits for PM_2.5_, PM_10_, NO_2_, SO_2_, and O_3_ are 25 μg/m^3^ (24-hour mean), 50 μg/m^3^ (24-hour mean), 200 μg/m^3^ (one-hour mean), 20 μg/m^3^ (24-hour mean), and 100 μg/m^3^ (eight-hour mean), respectively [9], while the limits for the same pollutants set by the USEPA are PM_2.5_ 35 μg/m^3^ (24-hour mean), PM_10_ 150 μg/m^3^ (24-hour mean), NO_2_ 100 ppb or 200 μg/m^3^ (one-hour mean), SO_2_ 75 ppb or 150 μg/m^3^ (one-hour mean) and O_3_ 0.075 ppm or 150 μg/m^3^ (one-hour mean) [10].

Data on the magnitude of air pollution in African cities is limited, particularly as it relates to Sub-Saharan Africa [11]. The WHO database provides an average PM_2.5_ value for Africa of 78 μg/m^3^ annual mean (which is almost three times the set limit) [12]. A detailed review of the database shows that 18 African studies, seven of which were from South Africa, were used in generating this average, indicating a dearth of data on air pollution for the African continent. In most of the African studies, PM concentrations exceed WHO limits. 

Data from African cities on gas phase pollutants are even sparser [11]. Available reports, however, indicate that concentrations of gas phase pollutants are low [13,14,15,16,17,18,19,20,21,22]. Carmichael *et al.*, in an extensive study in Africa, Asia and South America, found that concentrations of gas phase pollutants of SO_2_, NO_2_ and ammonia were generally lower in the tropical regions than non-tropical regions of the studied countries [23].

For Uganda, data on air pollution is nearly nonexistent. To date, there is only one publication available, from our group, showing a PM concentration of 100 μg/m^3^ from a single pilot study measured in one district of Kampala [24]. The 2010 Uganda State of the Environment Report acknowledges this lack of air pollution data for Uganda [25]. The current study expands on our previous air pollution assessment efforts [22] and provides novel data on ambient concentrations of four key air pollutants (PM_2.5_, NO_2_, SO_2_, and O_3_) at various sites in Kampala and Jinja. 

## 2. Methods

### 2.1. Study Design

This is a cross-sectional, spatial, pilot assessment of ambient air concentrations of PM_2.5_, NO_2_, SO_2_, and O_3_ at different sites in three different land use areas in Kampala and Jinja during the period from 30 June to 27 July, 2014.

### 2.2. Study Sites and Monitoring Approaches

Air pollutant monitoring was conducted in Kampala and Jinja. Kampala, the capital city of Uganda, covers an area of 197 km^2^ and is spread over 22 hills at an altitude of 1120 m above sea level. The city’s day and night population is 3 million and 1.72 million people, respectively [26]. The day population represents Kampala residents and commuters entering the city from outside regions for work, education and business. Annual rainfall in Kampala ranges from 1750–2000 mm with peak wet seasons from March to May and from September to November. The dry seasons are between June and July, and December and February. The average annual temperature is 21.9° C and relative humidity ranges from 53–89% [27].

Jinja is the second largest city in Uganda, located 80 km east of Kampala and covers a land area of 28 km^2^ at an altitude of 1230 m above sea level. Jinja has a day population of 300,000 people and a night population of 89,700 people [26]. The annual rainfall averages 1125 mm [28].

**Figure 1:**
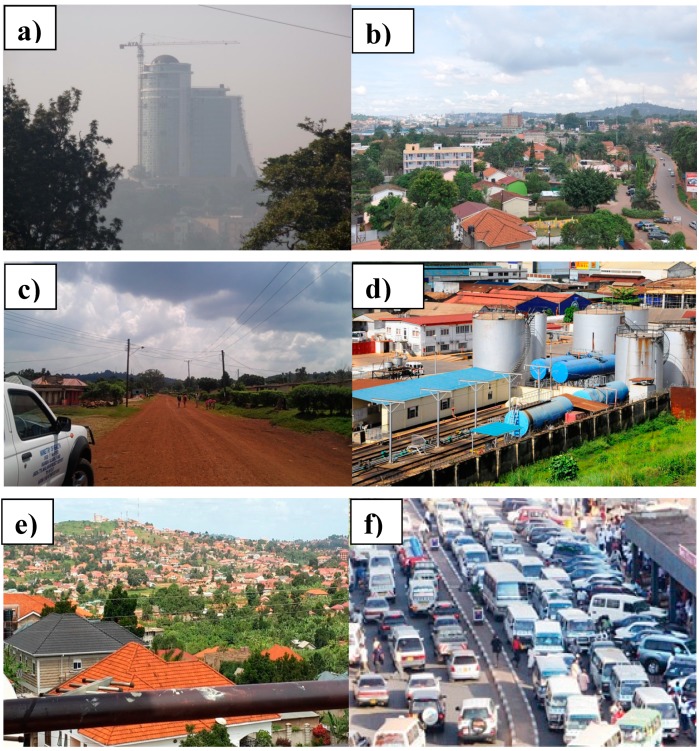
Representative images of sampling sites. Sampling site: **a**) central monitoring site; **b**) paved residential; **c**) unpaved residential, low income; **d**) industrial area; **e**) unpaved residential high income; and **f**) commercial center.

Air pollutant sampling sites in both cities were selected to represent different topographies and land use areas: commercial, industrial, and residential. According to the land classification system of local climate zones (LCZ), all of the sampling sites belong to the following categories: open low-rise or sparsely built [29]. Representative photographs of these sites are shown in Figure 1. Sites for PM monitoring were fewer than sites for gas phase pollutant monitoring, as equipment for PM sampling was limited.

Areas of the cities characterized by high commercial activities such as trading, small-scale manufacturing and high traffic were selected as commercial land use areas. Industrial land use areas were in designated industrial areas of the cities. In Kampala, the industries surrounding the monitoring sites were involved in textile, steel and food products, while in Jinja we observed food products, plastics and steel industries. Land use areas defined as residential were divided into two categories, those with paved/tarmac roads and those with unpaved/murram roads. 

Meteorological parameters (temperature and humidity) and O_3_ were monitored at one central commercial site in Kampala city. Meteorological data (2012–2014) were also retrieved from National Weather Services to compare the year-round meteorological conditions and the meteorological conditions during sampling.

### 2.3. Air Pollutant Sampling Methods

PM_2.5_ concentrations were measured over periods of 24 hours from 30 June to 27 July 2014 with a real-time aerosol monitor, DUSTTRACK II-8530 (TSI Inc, Shoreview, MN) at 18 sites (15 in Kampala and 3 in Jinja) that can assess PM_2.5_ concentrations in a range from 1 µg/m^3^ to 400 mg/m^3^. Prior to all measurements, the DUSTTRACK monitor was calibrated using the federal reference method, and zero-calibrated prior to each sampling session. All data were saved on the monitor until the end of study when it was downloaded into an excel database for analysis in Stata 11.2.

Concentrations of NO_2_ and SO_2_ were measured with Combo diffusion tubes (NO_2_ and SO_2_, Ormantine, FL, USA) at 28 study sites (22 in Kampala and 6 in Jinja). At each study site, two duplicate diffusion tubes were secured on the outside walls of selected buildings, 3–5 meters above ground. Sampling sites were selected to reflect different land use patterns and geographic topography, and each building was at least 3 meters away from immediate emission sources. The sampling height was selected for the safety of the passive samplers and was within the USEPA ambient monitoring siting criteria (*i.e.*, < 15 m). Each passive diffusion tube was exposed to ambient air for two weeks. Sampling starting and sampling end times were recorded. Two traveling blanks were included for each city. Prior to, and following, sampling periods, the samplers were stored at 4 °C. Combo diffusion tubes were shipped to Gradkos laboratory in England where NO_2_ and SO_2_ analyses were conducted on a Dionex ICS1100 ICU10 ion chromatography system (Thermo Fisher Scientific Inc., Waltham, MA, USA). O_3_ was measured with a federal equivalent real-time monitor (POM, 2B Technologies, CO, USA) that was calibrated before the study period, and cleaned daily during the sampling period. 

### 2.4. Meteorological Measurements

Temperature and humidity were monitored daily for the first seven days of the study period with a real-time monitor (HOBO U23, OnSet, MA, USA). The monitor was calibrated prior to sampling, and cleaned daily during the sampling period. 

### 2.5. Data Analysis

Data from all monitors were downloaded directly into a Microsoft Excel database and analyzed using Stata 11.2 (StataCorp LP, College Station, TX). Descriptive statistics were used to summarize all pollutant concentrations and meteorological data. Mean pollutant concentrations were compared between land use areas and cities by t-tests. A *p*-value of <0.05 was considered statistically significant. Concentrations of travelling blanks (*i.e.*, tubes not exposed to sampling environments) were subtracted from all measured SO_2_ and NO_2_ concentrations prior to statistical analysis.

### 2.6. Ethics Approval

The study protocol was approved by the Mulago Hospital Research and Ethics Committee and the Uganda National Council for Science and Technology.

## 3. Results 

### 3.1. Temperature and Humidity

Temperature and relative humidity were measured at the central monitoring location during the first week (30 June to 5 July, 2014) of sampling. The mean (± standard deviation (SD)) ambient temperature was 24.7 ± 1.9 °C (maximum 26.4 °C, minimum 21.1 °C). Mean humidity was 63.5% ± 5.7 (maximum 74.4%, minimum 58.0%). We did not monitor temperature and humidity data further due to limited equipment availability. Archived temperature and humidity data from the National Weather Services (at Entebbe Airport) was used instead and is presented in Table 1 showing largely constant weather conditions during the entire air pollution monitoring period. 

**Table 1:** Meteorological conditions during sampling days.

Sampling Date	Minimum Temperature °C	Maximum Temperature °C	Average Temperature °C	Average Relative Humidity %	Wind Speed Km/h	Precipitation mm
30 June	19	26	22	80	8	0.0
1 July	18	26	22	77	8	0.0
2 July	18	26	22	77	8	0.0
3 July	19	26	22	80	10	0.0
4 July	19	26	22	76	7	0.0
5 July	17	26	22	76	9	0.0
6 July	19	26	22	70	9	0.0
7 July	18	25	22	81	11	0.0
8 July	18	25	22	75	11	0.0
9 July	19	20	20	96	4	0.0
10 July	18	23	--*	--	--	--
11 July	17	24	20	76	11	0.0
12 July	20	23	22	84	10	0.0
13 July	19	25	22	76	9	0.0

* Data were missing, ** The historical minimum and maximum temperatures during the dry season are 18 and 28 °C for January, 18 and 28 °C for February, 17 and 25 °C for June, 17 and 25 °C for July, 16 and 25 °C for August, and 17 and 27 °C for December.

### 3.2. PM_2.5_

**Figure 2:**
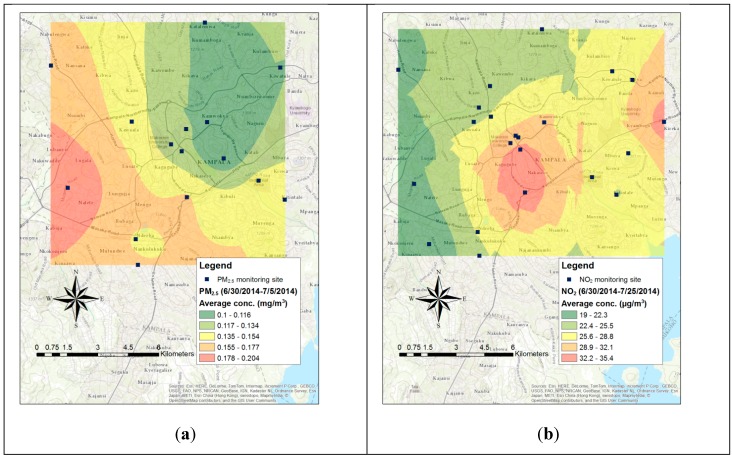
The spatial variation of PM_2.5_ (**a**) and NO_2_ (**b**) in Kampala.

PM_2.5_ concentrations were measured at 18 sites for an average sampling period of 21 hours and 15 minutes (maximum 24 hours, minimum 7 hours). Spatial variations of PM_2.5_ pollution levels in Kampala are shown in Figure 2a.

The PM_2.5_ concentrations by sampling site are presented in a supplementary Table S1. The mean 24-hour PM_2.5_ concentrations calculated for all study sites was 132.1 μg/m^3^. The concentration measured by the real-time monitor was comparable to the filter-based approach. At the central monitoring site (city center), the PM_2.5_ mass concentrations were 90.4 µg/m^3^ obtained from the filter-basedapproach*vs.* 94.0 µg/m^3^ obtained from the real-time monitor. By city, 24-hour mean PM_2.5_ concentrations in Kampala were higher than in Jinja, but the difference did not reach statistical significance (138.6 μg/m^3^ and 99.3 μg/m^3^, *p* = 0.20). By land use, PM_2.5_ and nitrogen dioxide pollution levels are shown in Table 2. The highest 24-hour mean PM_2.5_ concentrations were observed at the industrial (156 μg/m^3^) followed by residential areas with unpaved roads (152.6 μg/m^3^) and commercial land use areas (129.4 μg/m^3^). Residential and office areas with paved roads had the lowest mean PM concentrations (88.3 μg/m^3^). Compared to residential areas with paved roads, residential areas with unpaved roads had significantly higher mean 24-hour PM_2.5_ concentrations (152.6 μg/m^3^ *vs.* 88.3 μg/m^3^, *p* = 0.045). Considering both industrial and commercial areas as nonresidential, no significant differences were noted between nonresidential and residential areas (131.0 μg/m^3^ *vs.* 132.8 μg/m^3^, *p* = 0.93). The 24-hour mean PM_2.5_ concentration at a site in Jinja with unpaved roads was comparable to sites with unpaved roads in Kampala (161 μg/m^3^ *vs.* 151.4 μg/m^3^). The mean PM_2.5_ concentration at sites with paved roads in Jinja, however, was lower than that at similar sites in Kampala (68.5 μg/m^3^ *vs.* 108.0 μg/m^3^). 

### 3.3. Gas Phase Pollutants

Duplicate diffusion tubes were used for sampling SO_2_ and NO_2_ at each monitoring site. Each tube can simultaneously collect NO_2_ and SO_2_. The concentrations of NO_2_ and SO_2_ at each monitoring site were calculated as the average of the readings of the two tubes.

NO_2_ and SO_2_ concentrations were measured at a total of 28 monitoring sites (22 in Kampala and six in Jinja). In Kampala, one of the SO_2_ duplicate tubes could not be retrieved at two monitoring sites. In Jinja, both NO_2_ tubes could not be retrieved at one site and one SO_2_ tube only was retrieved at one site. Therefore, 27 NO_2_ and SO_2_ sampling tubes were available for analysis (22 from Kampala and five from Jinja). The mean monitor exposure time was 330.34 (±25.54) hours or 13 days and 19 hours. The overall precision, expressed as coefficient of variation based on 22 pairs of co located sampling, was 14.0%.

**Table 2:** PM_2.5_ and NO_2_ concentration by land use.

Land Use Category	PM_2.5_			NO_2_	
	Mean (SD) 24 Hour Average	Mean (SD) Minimum	Mean (SD) Maximum	Mean (SD) NO_2_ (µg/M^3^)	Mean (SD) NO_2_ (Ppb)
**Commercial Area**	129.4 (38)	4.82 (31)	284.4 (89)	32.19 (12.19)	16.79 (6.49)
**Industrial Area**	156 (0)	8.2 (0)	384 (0)	22.69 (5.76)	11.76 (2.99)
**Residential Unpaved (Murram) Road**	152.6 (44)	23.1 (35)	346.1 (95)	20.09 (5.67)	11.61 (4.88)
**Residential/Office Paved (Tarmac)**	88.3 (50)	3.9 (27)	155 (66)	18.39 (4.39)	11.43 (3.16)

#### 3.3.1. Nitrogen Dioxide

NO_2_ concentrations determined at the different sampling sites are shown in supplementary Table S2. The mean two-week NO_2_ concentration was 24.9 µg/m^3^.

By city, Kampala air was characterized by a higher mean total NO_2_ concentration than that of Jinja (26.69 µg/m^3^ *vs.*17.49 µg/m^3^, *p* = 0.07). The spatial variations of NO_2_ concentration levels in Kampala are shown in Figure 2b.

NO_2_ concentrations by land use are shown in Table 2. The highest NO_2_ concentrations were observed in commercial land use areas (32.19 µg/m^3^) and the lowest in residential land use areas with paved roads (18.39 µg/m^3^). The mean NO_2_ concentrations in commercial (including industrial) land use areas were significantly higher than in residential land use areas (32.19 µg/m^3^ *vs.* 19.69 µg/m^3^, *p* = 0.002). NO_2_ concentrations measured in residential land use areas did not significantly differ from those in industrial land use areas (19.69 µg/m^3^ *vs.* 22.69 µg/m^3^, *p* = 0.46). Similarly, NO_2_ concentrations in commercial land use areas did not significantly differ from those in industrial land useareas(32.19µg/m^3^*vs.* 22.69 µg/m^3^, *p* = 0.22).

#### 3.3.2. Sulfur Dioxide

SO_2_ was measured at all study sites and in all land use areas where NO_2_ was measured. Levels of detectable SO_2_ concentrations are shown in supplementary Table S3. SO_2_ concentrations were below detection limit (<0.03 µg/m^3^) at 20 of the 27 monitoring sites. The two-week mean SO_2_ concentration at all monitoring sites was 3.79 ± 3.0 µg/m^3^. 

By city, there was only one monitoring site in Jinja where SO_2_ concentrations reached detectable levels. This site, in an industrial land use area of Jinja, had a higher SO_2_ concentration level (7.3 µg/m^3^) than that in the industrial land use area of Kampala (<0.69 µg/m^3^). Six other monitoring sites in Kampala also showed SO_2_ concentrations above detection limit (mean 3.11 µg/m^3^). Comparing SO_2_ concentrations by land use, concentrations were highest in industrial land use areas (7.39 µg/m^3^), followed by commercial land use areas (3.69 µg/m^3^), then residential land use areas with paved roads (2.79 µg/m^3^), or unpaved roads (2.39 µg/m^3^).

#### 3.3.3. Ozone

**Figure 3:**
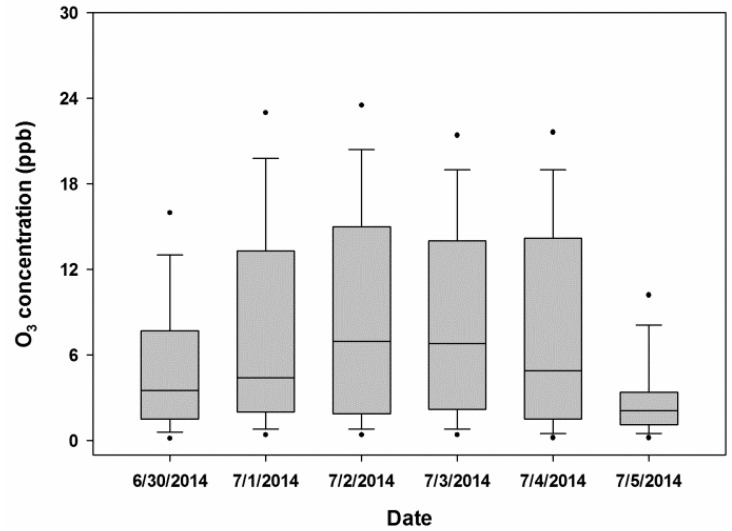
Distribution of O_3_ concentrations (1-min average) measured at the central monitoring site in Kampala.

The mean one-hour O_3_ concentration at the monitoring site established at Lourdel Road, Wandegeya in Kampala measured over a period of seven days was 11.4 μg/m^3^ (±4.8 μg/m^3^). O_3_ concentrations varied considerably across the sampling period (Figure 3).

## 4. Discussion

This pilot study demonstrates presence of high PM concentrations and low gas phase air pollutant levels in Kampala and Jinja between 30 June and 17 July, 2014. The observed mean PM_2.5_ concentration of 132.1 μg/m^3^ (5.3-fold above the limit defined by WHO) across all monitoring sites in the current study is comparable with the mean PM_2.5_ concentration of 104.9 μg/m^3^ described in an earlier single-site pilot study from a district in Kampala [24]. As expected, particulate air pollution levels were found to be greatest in areas with high commercial/industrial land use and unpaved roads. 

The observed mean PM_2.5_ concentration of 132.1 μg/m^3^ in the current study clearly exceeds the mean PM concentration of 78 μg/m^3^ calculated for the African region and reported in the WHO 2014 publication of the global state of air quality from 1600 cities in 91 countries [12]. Within the East African region, however, the mean PM concentration (132.1 μg/m^3^) observed in the current study is comparable with that reported from Nairobi/Kenya (128.7 μg/m^3^) and significantly higher than that reported from Dar es Salaam/Tanzania (26 μg/m^3^) [17,30]. 

Sources of particulate air pollution described in the studies of African cities are typically emissions from vehicles, re-suspended dust from unpaved roads, smoke from indoor biomass fuel use and garbage burning, and industrial sites [14,31,32]. During the current study, we observed source emissions of dust and soil blown by wind from unpaved roads, black smoke exhausts from cars, trucks and busses and smoke from burning household garbage in both Kampala and Jinja. High PM levels in residential land use areas with unpaved roads without industrial activity or high traffic volume, suggest that re-suspended dust significantly contributes to high PM levels. High PM levels in commercial land use areas with high traffic volume and paved roads, in contrast, suggest that vehicle emissions represent another significant source of PM in Kampala and Jinja. Dust from unpaved roads in the suburbs of both cities appears to be carried by human activities into areas with paved roads. 

Ambient air PM composition has been reported in some African cities [16,24,33]. Our earlier pilot study in Kampala found that more than 90% of PM_2.5_ studied at a sampling site in the Mpererwe district of Kampala was comprised of crustal species (probably re-suspended soil dust) and carbonaceous aerosol [24]. In Dar es Salaam, a study of PM collected close to a vehicle traffic site found carbon to be the main component suggesting vehicular emissions as its main source [33]. In Nairobi, Gaita *et al.* found that vehicle traffic, mineral dust, industrial activity, combustion and a mixed factor (composed of biomass burning, secondary aerosol and aged sea salt) were the main sources of PM air pollution [32]. Mineral dust and traffic were responsible for approximately 74% of PM_2.5_ mass [32]. Based on our findings and observations in the current pilot study we speculate that re-suspended dust and vehicular emissions are the primary sources of PM_2.5_ in Kampala and Jinja and may also be significant contributors to air pollution in other African cities.

We also assessed the concentration of three key gas phase pollutants (NO_2_, SO_2_ and O_3_) in Kampala and Jinja. Even though concentrations of NO_2_, SO_2_ and O_3_ were below WHO guideline levels (200 μg/m^3^ one-hour mean, 20 μg/m^3^ 24-hour mean and 100 μg/m^3^ eight-hour mean, respectively [2]) we recognize that our findings cannot be directly compared with WHO air quality standards due to differences in averaging times. However, our findings are comparable with findings from other gas phase pollutant studies in Africa [13,14,15,17]. We do not know why gas phase pollutants in our study and other studies from Africa are low. Climatic conditions in the studied areas may facilitate adsorption of gas phase pollutants onto PM. As PM concentrations were found to be high in the current study and other studies in Africa this may explain the observed low concentrations of gas phase pollutants.

Conclusions from the current cross sectional study have to be made considering that temporal variations of air pollutant concentrations could not be assessed, which is a major limitation of this pilot study, especially for PM_2.5_. Due to the limitation of the number of real-time instruments (*i.e.*, only one DustTrack), we could not measure PM_2.5_ concentrations at multiple sites at the same time. Therefore, we were unable to differentiate spatial variation from temporal variation in PM_2.5_ concentrations. However, the meteorological data in Table 1 suggests that the weather conditions during our measurements were quite consistent, minimizing the possibility of the impact of weather on PM_2.5_ concentrations. Given that this study was conducted within a short period of time, source emission profiles in different locations will not be expected to change dramatically. Therefore, the measured PM_2.5_ spatial variation at minumum suggests the heterogeneous pattern of PM_2.5_ in Kampala.

In addition, PM_2.5_ concentrations (132.1 μg/m^3^) observed in this study (dry season) are consistent with findings from our earlier study, in which PM_2.5_ (104.3 μg/m^3^) was collected in December 2013 (also a dry season). Meteorological conditions during this pilot study were typical in Kampala for dry seasons, as shown in Table 1. We are aware of the impact of weather and seasonal variations on air pollutant concentrations and expect PM_2.5_ concentrations to be different in rainy seasons. Future studies will have to expand air pollution monitoring to other cities and parts of Uganda, cover all weather seasons and begin exploring air pollution effects on public health, in particular lung health in urban populations of Uganda.

## 5. Conclusions

This study suggests that high level PM air pollution is prevalent in urban and suburban areas in Uganda, with PM_2.5_ concentrations above 100 µg/m^3^ in multiple locations in Kampala. Land use characteristics define ambient PM_2.5_, NO_2_ and SO_2_ concentrations. Long-term exposures to the observed high levels of air pollution likely represent a major risk to public health in Kampala and Jinja. Long-term studies are needed to assess air pollution levels during the course of multiple weather seasons and the health impact in exposed populations.

## Supplementary Tables

**Table S1:** PM_2.5_ concentrations at different sites in Kampala and Jinja.

Site	City	Land Use	Test Length (H: Min)	PM _2.5_ Concentration, µg/M^3^			Time Min (H:Min:S)	Time Max (H:Min:S)
				24 hour Average	Min	Max		
Bugolobi	Kampala	Residential/office paved (tarmac) road	24:00	163	66	254	16:18	23:18:48
Kampala Industrial Area	Kampala	Industrial area	23:35	156	82	384	16:04:46	23:04:46
Kisimira Road	Jinja	Residential/office paved (tarmac) road	24:00	68	39	119	6:02:42	21:02:42
Rippon garden Nile Avenue	Jinja	Residential/office paved (tarmac) road	24:00	69	50	124	16:04:15	8:04:15
School village Tenywa Road Walukuba East	Jinja	Residential unpaved (murram) road	18:46	161	0	285	11:45:21	18:45:21
Kabowa Gabunga Road	Kampala	Residential unpaved (murram) road	24:00	156	40	362	14:45:47	7:45:47
Kamwokya, Kyebando Road	Kampala	Commercial area	24:00	88	0	217	13:05:53	14:05:53
Katanga, Wandegeya	Kampala	Commercial area	24:00	108	52	248	6:56:31	18:56:31
Kawala Bwaise Road	Kampala	Residential unpaved (murram) road	23:35	135	0	291	13:54:31	21:54:31
Kiwatule Central 1	Kampala	Residential unpaved (murram) road	7:00	114	90	22	17:43:30	20:43:30
Kololo Ekobo Road	Kampala	Residential/office paved (tarmac) road	18:00	53	2	123	17:43:28	10:43:28
Kyanja Nazareth Road	Kampala	Residential unpaved (murram) road	24:00	100	55	317	11:15:43	21:15:43
Lungujja Busega Kibumbiro	Kampala	Residential unpaved (murram) road	22:09	240	0	535	7:38:18	8:38:18
Mulago Mulago Hill Road	Kampala	Commercial area	24:00	143	59	225	9:43:39	2:43:39
Amir street Nakasero	Kampala	Commercial area	19:00	187	85	434	16:14:20	2:14:20
Nalukolongo Kweba Zone	Kampala	Residential unpaved (murram) road	18:16	133	0	401	9:28:42	0:28:42
Nansana Naluvule	Kampala	Residential unpaved (murram) road	22:32	182	0	358	14:26:25	23:26:25
Lourdel Road, wandegeya	Kampala	Commercial area	17:00	121	45	298	21:57:34	1:57:34

**Table S2:** Concentration of ambient air nitrogen dioxide at different sites in Kampala and Jinja cities.

Site	Land Use	City	Exposure	NO_2_, total (µg)	NO_2_, µg/m^3^	NO_2_ ppb
Amir Street–Nakasero	Commercial center	Kampala	309.55	1.15	49.90	26.96
Mutundwe–Kigaga Zone	Residential unpaved (murram) road	Kampala	337.75	0.25	9.32	4.85
Nalukolongo–Kweba Zone	Residential unpaved (murram) road	Kampala	337.67	0.67	26.22	13.64
Kawala–Bwaise Road	Residential unpaved (murram) road	Kampala	339.50	0.62	24.33	12.66
Kiwat ule Central I.	Residential unpaved (murram) road	Kampala	336.00	0.56	22.08	11.48
Bwaise–Makerere–Kavule Road	Residential unpaved (murram) road	Kampala	308.43	0.54	23.33	12.13
Bwaise, x Road	Commercial center	Kampala	337.00	0.61	24.15	12.56
Kabowa–Gabunga Road	Residential unpaved (murram) road	Kampala	338.07	0.59	23.00	11.96
Lungunja–Busega–Kibumbiro	Residential unpaved (murram) road	Kampala	335.00	0.50	19.71	10.25
Rippon Garden–Nile Avenue	Residential/office paved (tarmac) road	Jinja	337.07	0.49	19.25	10.01
School Village–Tenywa Road Walukuba East	Residential unpaved (murram) road	Jinja	336.83	0.27	10.48	5.45
Walukuba - Masese Road	Commercial center	Jinja	336.42	0.29	11.14	5.79
Mulago Hill Road 1	Commercial center	Kampala	343.55	0.59	22.79	11.85
Mulago Hill Road 2	Commercial center	Kampala	311.63	0.78	31.78	16.53
Bugolobi	Residential/office paved (tarmac) road	Kampala	336.63	0.39	15.23	7.92
Banda Zone B8–Banda Nalya Road	Commercial center	Kampala	332.48	0.68	27.22	14.15
Namugongo Road, Kireka	Commercial center	Kampala	332.45	1.31	52.90	27.51
Katanga, Wandegeya	Commercial center	Kampala	331.50	0.81	32.72	17.02
Kawempe–Mbogo Road	Residential/office paved (tarmac) road	Kampala	333.50	0.61	24.14	12.55
Nansana–Nalvule	Residential unpaved (murram) road	Kampala	332.42	0.53	21.26	22.70
Mbuya–Nadiope	Residential/office paved (tarmac) road	Kampala	215.05	0.24	14.63	15.22
Lourdel road, Wandegeya	Commercial center	Kampala	360.00	1.13	42.08	21.88
Kamwokya–Kyebando Road	Commercial center	Kampala	336.92	0.79	31.22	16.23
Kyanja–Nazereth Rd/Kyanja Road	Residential unpaved (murram) road	Kampala	356.12	0.57	21.19	11.02
5th Street Industrial Area	Industrial area	Kampala	334.02	0.67	26.67	13.87
Nizam Road, Jinja center	Commercial center	Jinja	335.50	0.69	27.37	14.23
Industrial Area—Jinja	Industrial area	Jinja	338.15	0.47	18.53	9.64

**Table S3:** Concentration of Sulphur dioxide at sites with detectable Sulphur dioxide concentrations.

Site	Land Use	City	Exposure	Total SO_2_ (µg)	SO_2_ (µg/m^3^)	SO_2_ (ppb)
Kabowa-Gabunga Road	Residential unpaved (murram) road	Kampala	338.07	0.05	2.34	0.88
Mulago Hill road 1	Commercial centre	Kampala	311.63	0.04	1.73	0.65
Bugolobi	Residential/office paved (tarmac) road	Kampala	336.63	0.04	1.81	0.68
Banda Zone B8-Banda Nalya Road	Commercial centre	Kampala	332.48	0.1	8.35	3.13
Namugongo Road-Kireka road	Commercial centre	Kampala	332.45	0.03	0.77	0.29
Kawempe-Mbogo Road	Residential/office paved (tarmac) road	Kampala	333.5	0.06	3.65	1.37
Industrial Area-Jinja	Industrial area	Jinja	338.15	0.09	7.31	2.74

## References

[1] 7 Million Premature Deaths Annually Linked to Air Pollution. http://www.who.int/mediacentre/news/releases/2014/air-pollution/en/.

[2] World Health Organisation Health Topics. Air Pollution. http://www.who.int/topics/air_pollution/en/.

[3] Environmental Working Group Body Burden–—The Pollution in Newborns. A Benchmark Investigation of Industrial Chemicals, Pollutants and Pesticides in Umbilical Cord Blood. http://www.ewg.org/research/body-burden-pollution-newborns.

[4] Salvi S. (2007). Health effects of ambient air pollution in children. Paediatr. Respir. Rev..

[5] Kajekar R. (2007). Environmental factors and developmental outcomes in the lung. Pharmacol. Ther..

[6] Fleischer N.L., Merialdi M., van Donkelaar A., Vadillo-Ortega F., Martin R.V., Betran A.P., Souza J.P., O’Neill M. (2014). Outdoor air pollution, preterm birth, and low birth weight: analysis of the world health organization global survey on maternal and perinatal health. Environ. Health Perspect..

[7] Fenger J. (1999). Urban air quality. Atmospheric Environ..

[8] Clean Air Act. http://www.epw.senate.gov/envlaws/cleanair.pdf.

[9] World Health Organization (2006). WHO Air Quality Guidelines for Particulate Matter, Ozone, Nitrogen Dioxide and Sulfur Dioxide: Global Update 2005: Summary Of Risk Assessment.

[10] National Ambient Air Quality Standards (NAAQS). http://www.epa.gov/air/criteria.html.

[11] Petkova E.P., Jack D.W., Volavka-Close N.H., Kinney P.L. (2013). Particulate matter pollution in African cities. Air Quality Atmos. Health.

[12] Ambient (Outdoor) Air Pollution in Cities Database 2014. http://www.who.int/phe/health_topics/outdoorair/databases/cities/en/.

[13] Khoder M.I. (2009). Diurnal, seasonal and weekdays-weekends variations of ground level ozone concentrations in an urban area in greater Cairo. Environ. Monit. Assess..

[14] Arku R.E., Vallarino J., Dionisio K.L., Willis R., Choi H., Wilson J.G., Hemphill C., Agyei-Mensah S., Spengler J.D., Ezzati M. (2008). Characterizing air pollution in two low-income neighborhoods in Accra, Ghana. Sci Total Environ..

[15] Moodley K.G., Singh S., Govender S. (2011). Passive monitoring of nitrogen dioxide in urban air: A case study of Durban metropolis, South Africa. J. Environ. Manage..

[16] Etyemezian V., Tesfaye M., Yimer A., Chow J., Mesfin D., Nega T., Nikolich G., Watson J.G., Wondmagegn M. (2005). Results from a pilot-scale air quality study in Addis Ababa, Ethiopia. Atmos. Environ..

[17] Jackson M.M. (2005). Roadside concentration of gaseous and particulate matter pollutants and risk assessment in Dar-es-Salaam, Tanzania. Environ. Monit. Assess..

[18] Lindén J. (2012). Intra-urban air pollution in a rapidly growing Sahelian city. Environ. Int..

[19] Josipovic M., Annegarn H.J., Kneen M.A., Pienaar J.J., Piketh S.J. (2010). Concentrations, distributions and critical level exceedance assessment of SO_2_, NO_2_ and O_3_ in South Africa. Environ. Monit. Assess..

[20] Kilabuko J.H., Matsuki H., Nakai S. (2007). Air quality and acute respiratory illness in biomass fuel using homes in Bagamoyo, Tanzania. Int. J. Environ. Res. Public Health.

[21] El-Dars F.M.S. (2004). Monitoring ambient sulfur dioxide levels at some residential environments in the Greater Cairo urban Region—Egypt. Environ. Monit. Assess..

[22] Adon M., Glay-Lacaux C., Yoboue V., Delon C., Lacaux J.P., Castera P., Gardrat E., Pienaar J., Al Ourabi H., Laouali D. (2007). Long term measurements of sulfur dioxide, nitrogen dioxide, ammonia, nitric acid and ozone in Africa using passive samplers. South Afr. J. Sci..

[23] Carmichael G.R., Ferm M., Thongboonchoo N., Woo J.-H., Chan L., Murano K., Viet P.H., Mossberg C., Bala R., Boonjawat J. (2003). Measurements of sulfur dioxide, ozone and ammonia concentrations in Asia, Africa, and South America using passive samplers. Atmos. Environ..

[24] Schwander S., Okello C.D., Freers J., Chow J.C., Watson J.G., Corry M. (2014). Ambient particulate matter air pollution in Mpererwe District, Kampala, Uganda: A pilot study. J. Environ. Public Health.

[25] State of the Environment Report For Uganda 2010. http://library.health.go.ug/publications/service-delivery-public-health/environment-and-sanitation/state-environment-report.

[26] Uganda Statistical Abstract 2012. http://www.ubos.org/onlinefiles/uploads/ubos/pdf%20documents/2012StatisticalAbstract.pdf.

[27] District Profile. http://ww2.unhabitat.org/programmes/ump/documents/kampala_cds.doc.

[28] Jinja Municipality Profile. http://www.skelleftea.se/Skol%20och%20kulturkontoret/Innehallssidor/Bifogat/JINJA%20MUNICIPALITY%20PROFILE.pdf.

[29] Stewart I.D., Oke T.R. (2012). Local climate zones for urban temperature studies. Bulletin Amer. Meteorol. Soc..

[30] Kinney P.L. (2011). Traffic impacts on PM(_2.5_) air quality in Nairobi, Kenya. Environ. Sci. Policy.

[31] Ofosu F.G., Hopke P.K., Aboh I.J., Bamford S.A. (2013). Biomass burning contribution to ambient air particulate levels at Navrongo in the Savannah zone of Ghana. J. Air Waste Manag. Assoc..

[32] Gaita S., Boman J., Gatari M., Pettersson J., Janhäll S. (2014). Source apportionment and seasonal variation of PM 2.5 in a Sub-Saharan African city: Nairobi, Kenya. Atmos. Chem. Phys..

[33] Mkoma S.L., Chi X., Maenhaut W. (2010). Characteristics of carbonaceous aerosols in ambient PM_10_ and PM_2.5_ particles in Dar es Salaam, Tanzania. Sci Total Environ..

